# Supplementary material for: Timing of Medium-Chain Triglyceride Consumption Modulates Effects in Mice with Obesity Induced by a High-Fat High-Sucrose Diet
Source: Nutrients. 2022 Dec 1;14(23):5096. doi: 10.3390/nu14235096 (PMC9740693; doi:10.3390/nu14235096)
Supplement: Supplementary file 1 [file nutrients-14-05096-s001.zip › nutrients-1994658-supplementary.pdf]

Supplementary information

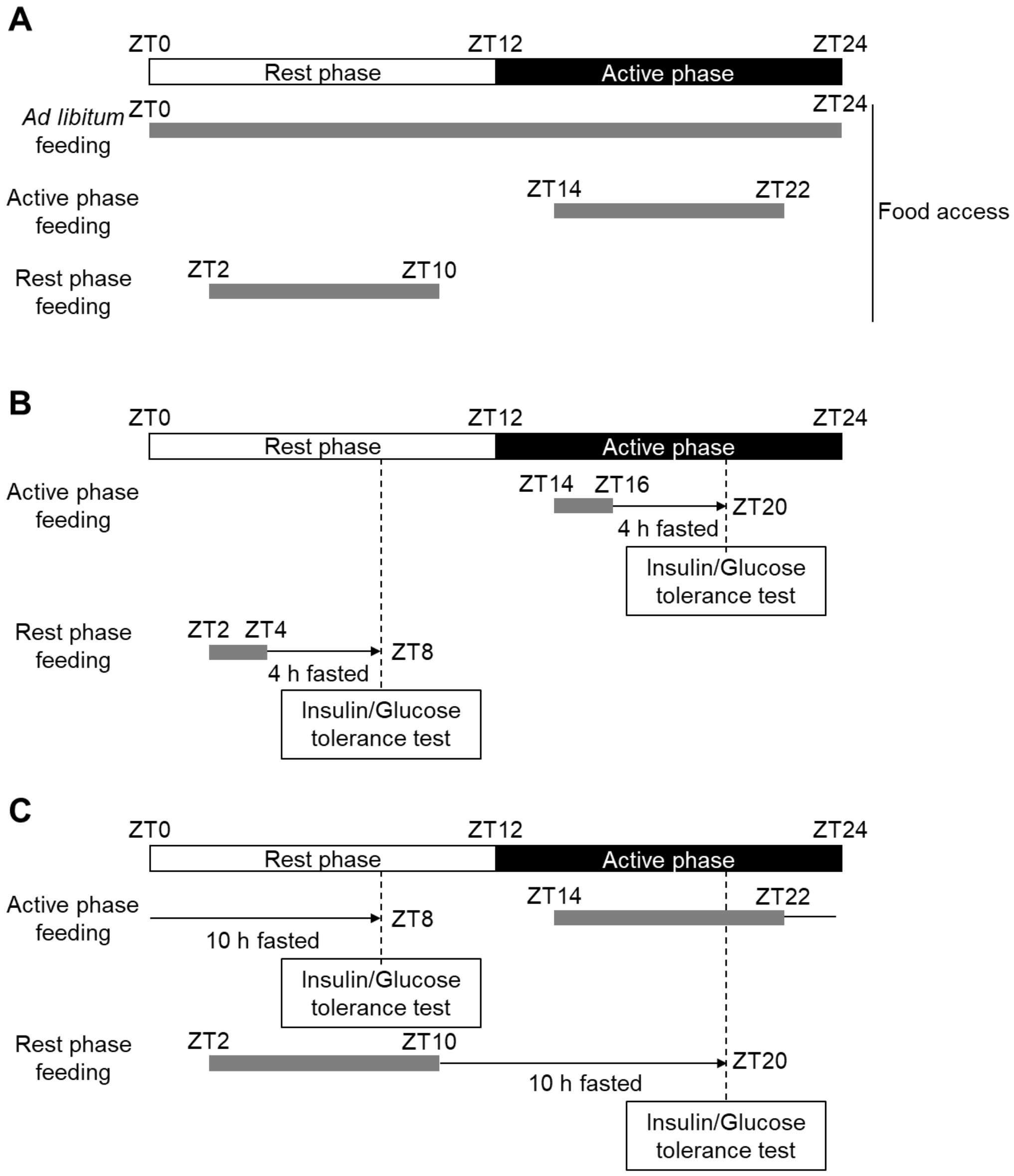

Supplementary Figure S1. Experimental time-restricted feeding and insulin/glucose tolerance test.

(A) Protocol for timing daily food access per 24 h. White and black bars, rest (light) and active (dark) phases, respectively. Protocol for timing insulin or glucose tolerance tests during feeding (B) or fasting (C) periods ZT, zeitgeber time.

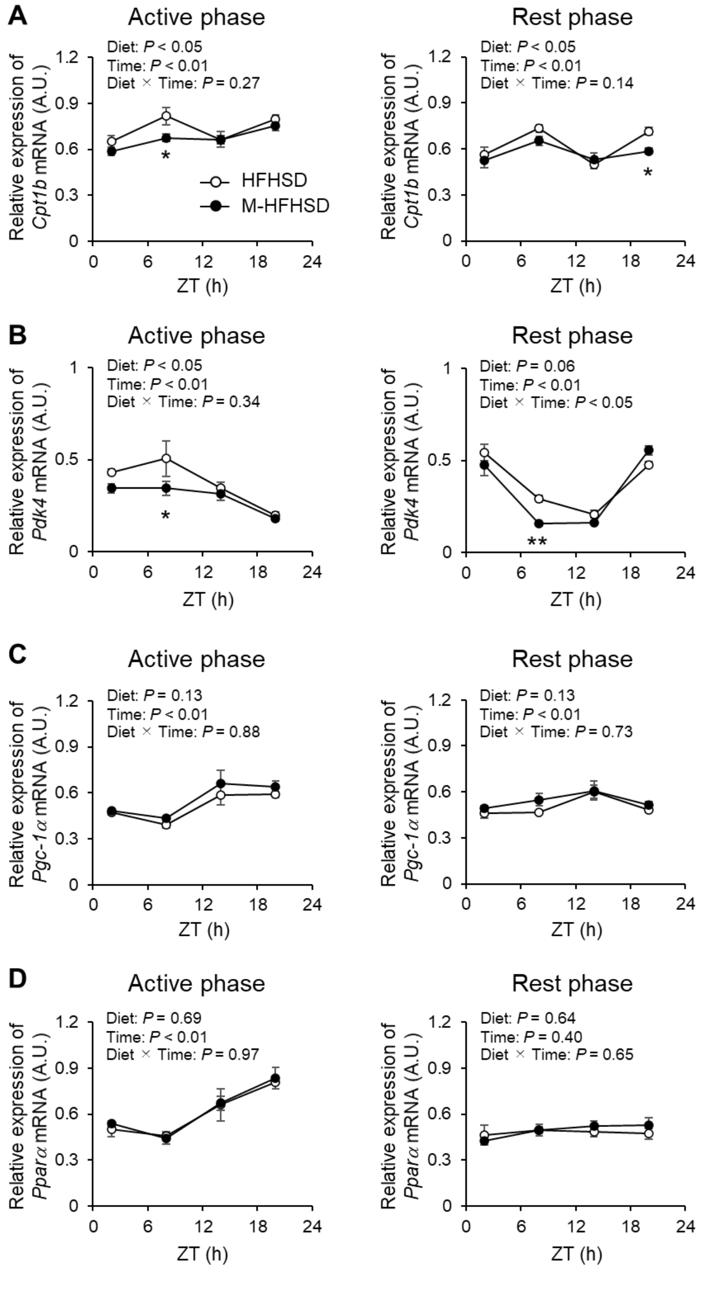

Supplementary Figure S2. Expression of genes associated with fatty acid oxidation in gastrocnemius muscle.

Expression of *Cpt1b* (A), *Pdk4* (B), *Pgc-1α* (C) and *PPARα* ((D) mRNA at indicated times in gastrocnemius muscles of mice fed only during active or rest phases. SEM (n = 4). *$P < 0.05$ and

**$P < 0.01$ *vs.* HFHSD at same time. Cpt1b, carnitine palmitoyltransferase 1b; HFHSD, high-fat high-sucrose diet; MCT, medium-chain triglyceride; M-HFHSD, HFHSD containing medium-chain triglyceride; Pdk4, pyruvate dehydrogenase kinase 4; Pgc-1α, peroxisome proliferator-activated receptor-coactivator-1α; PPARα, peroxisome proliferator-activated receptor; ZT, zeitgeber time.

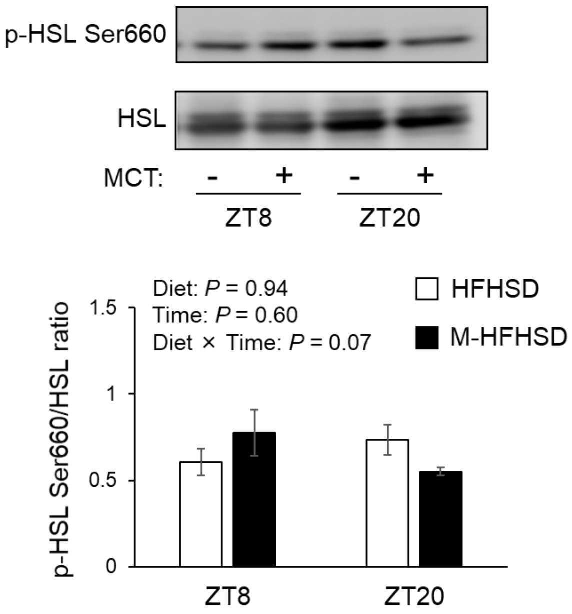

Supplementary Figure S3. Phosphorylation of hormone-sensitive lipase on Ser660 in epididymal white adipose tissue (eWAT) from mice fed *ad libitum*.

Protein expression of phosphorylated HSL on Ser660 and total HSL in eWAT extracts from mice. Ratios of phosphorylated protein-to-total protein. Data are means ± SEM (n = 4). eWAT, epididymal white adipose tissue; HFHSD, high-fat high-sucrose diet; HSL, hormone-sensitive lipase; MCT, medium-chain triglyceride; M-HFHSD, HFHSD containing medium-chain triglyceride; ZT, zeitgeber time.

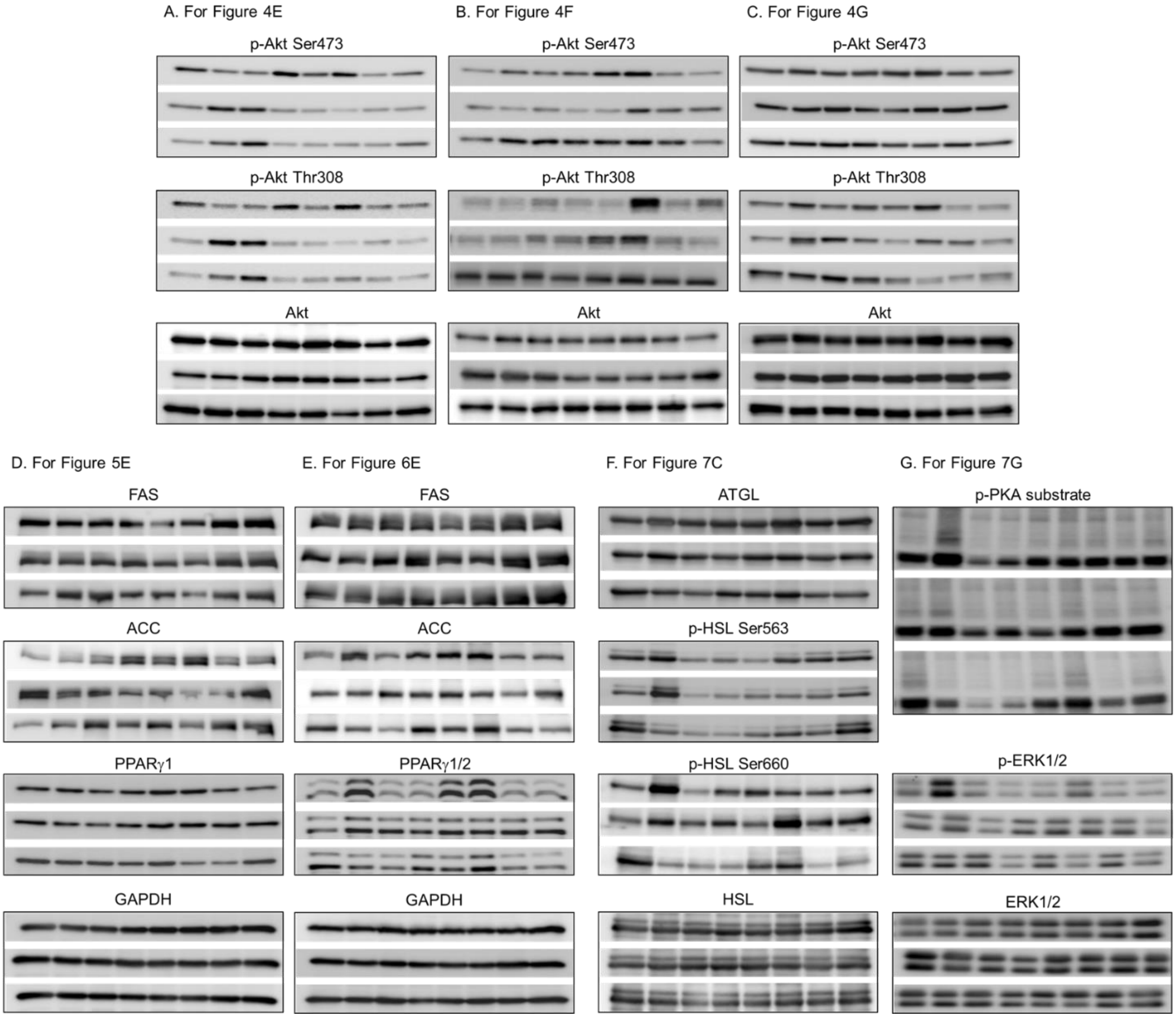

Supplementary Figure S4. All images of western blot used for quantification of densities.

Supplemental Table S1. Forward and reverse (5′ → 3′) primers for real-time RT-PCR.

Gene	Forward	Reverse
Acc1	ATTGGGCACCCCAGAGCTA	CCCGCTCCTTCAACTTGCT
Atgl	CTTGAGCAGCTAGAACAATG	GGACACCTCAATAATGTTGGC
Cpt1b	CCCATGTGCTCCTACCAGAT	CCTTGAAGAAGCGACCTTTG
Fas	GGAGGTGGTGATAGCCGGTAT	TGGGTAATCCATAGAGCCCAG
Hsl	GCTGGAGGAGTGTTTTTTTGC	AGTTGAACCAAGCAGGTCACA
Pdk4	CACATGCTCTTCGAACTCTTCAAG	TGATTGTAAGGTCTTCTTTTCCCAAG
Pgc-1a	GTAGGCCCAGGTACGACAGC	GCTCTTTGCGGTATTCATCCC
Pparα	TGCAAACTTGGACTTGAACG	AGGAGGACAGCATCGTGAAG
Pparg1	GTGAACCACTGATATTCAGGACAT	CCACAGAGCTGATTCCGAAGTTT
Pparg2	AACTCTGGGAGATTCTCCTGTTGA	TGGTAATTTCTTGTGAAGTGCTCATA
Srebp1c	ATCGGCGCGGAAGCTGTCGGGGTAGCGTC	ACTGTCTTGGTTGTTGATGAGCTGGAGCAT
36b4	CTTCATTGTGGGAGCAGACA	TCTCCAGAGCTGGGTTGTTC
Gapdh	ACCCAGAAGACTGTGGATGG	TTCAGCTCTGGGATGACCTT

Acc1, acetyl-CoA carboxylase 1; Atgl, adipose triglyceride lipase; Cpt1b, carnitine palmitoyltransferase 1b; Fas, fatty acid synthase; Hsl, hormone-sensitive lipase; Pdk4, pyruvate dehydrogenase kinase 4; Pgc-1α, peroxisome proliferator-activated receptor γ-coactivator-1α; Ppar, peroxisome proliferator-activated receptor; Srebp1c, sterol regulatory element-binding protein 1c; Gapdh, glyceraldehyde-3-phosphate dehydrogenase.
